# Structural and dynamical description of the enzymatic reaction of a phosphohexomutase

**DOI:** 10.1063/1.5092803

**Published:** 2019-04-01

**Authors:** Kyle M. Stiers, Abigail C. Graham, Jian-She Zhu, David L. Jakeman, Jay C. Nix, Lesa J. Beamer

**Affiliations:** 1 Biochemistry Department, University of Missouri, 117 Schweitzer Hall, Columbia, Missouri 65211, USA; 2 College of Pharmacy, Dalhousie University, 5968 College Street, Halifax, Nova Scotia B3H 3J5, Canada; 3 Department of Chemistry, Dalhousie University, Halifax, Nova Scotia B3H 4R2, Canada; 4 Molecular Biology Consortium, Advanced Light Source, Lawrence Berkeley National Laboratory, Berkeley, California 94720, USA

## Abstract

Enzymes are known to adopt various conformations at different points along their catalytic cycles. Here, we present a comprehensive analysis of 15 isomorphous, high resolution crystal structures of the enzyme phosphoglucomutase from the bacterium *Xanthomonas citri*. The protein was captured in distinct states critical to function, including enzyme-substrate, enzyme-product, and enzyme-intermediate complexes. Key residues in ligand recognition and regions undergoing conformational change are identified and correlated with the various steps of the catalytic reaction. In addition, we use principal component analysis to examine various subsets of these structures with two goals: (1) identifying sites of conformational heterogeneity through a comparison of room temperature and cryogenic structures of the apo-enzyme and (2) *a priori* clustering of the enzyme-ligand complexes into functionally related groups, showing sensitivity of this method to structural features difficult to detect by traditional methods. This study captures, in a single system, the structural basis of diverse substrate recognition, the subtle impact of covalent modification, and the role of ligand-induced conformational change in this representative enzyme of the α-D-phosphohexomutase superfamily.

## INTRODUCTION

**FIG. 1.**
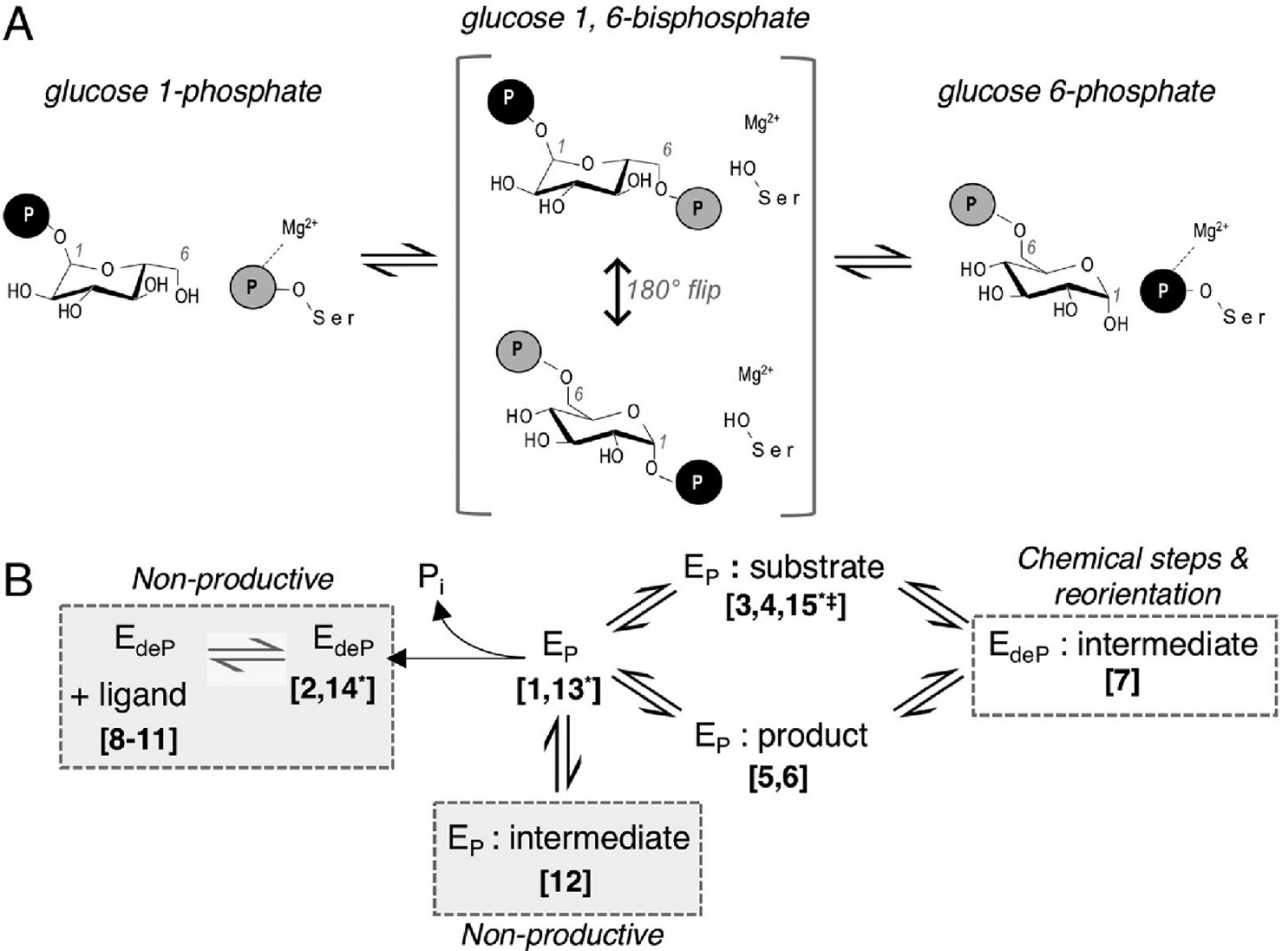
Overview of the mechanism and structure of XcPGM. (a) A schematic of the catalytic reaction, showing the reversible conversion of glucose 1-phosphate to glucose 6-phosphate. Glucose 1,6-bisphosphate undergoes a 180° reorientation in between the two phosphoryl transfer steps of the reaction. (b) An outline of the catalytic cycle of XcPGM, highlighting the various enzyme states captured in this study. Structure numbers correspond to those on Table I. Structures not part of the normal catalytic cycle are labeled as “nonproductive” and highlighted by gray boxes. Structures previously determined in another study^3^ are analogous to 1, 3, and 11. ^*^Room-temperature data set collected; **^‡^**complex with a nonhydrolysable G1P analog (see the text).

The α-D-phosphohexomutases are ubiquitous enzymes found in all kingdoms of life.^1^ Among other reactions, these enzymes catalyze the reversible conversion of 1-phospho to 6-phosphohexoses, with various sugars including glucose, mannose, glucosamine, and N-acetylglucosamine. These reactions are fundamental in carbohydrate metabolism, required for processes such as glycogen synthesis and breakdown, and protein glycosylation. In bacteria, the enzymes phosphoglucomutase (PGM) and phosphomannomutase/phosphoglucomutase (PMM/PGM) are involved in the biosynthesis of exopolysaccharides that contribute to the pathogenicity of infections in higher organisms, including humans, animals, and plants.^1–3^ The reaction mechanism of the α-D-phosphohexomutases is conserved, and entails two phosphoryl transfers [Fig. 1(a)], with the initial transfer occurring from a phosphoserine residue of the enzyme to a monophosphorylated sugar to form a bisphosphorylated intermediate (e.g., glucose 1,6-bisphosphate or G16P). This is followed by the second phosphoryl transfer from the alternate phospho-group of the intermediate back to the enzyme, creating product and regenerating the active, phosphorylated state of the enzyme.

The multistep reaction of the α-D-phosphohexomutases has several unique features that pose considerable challenges for macromolecular recognition. One of these is that the enzyme utilizes the same catalytic residues for phosphoryl transfer to both the 1- and 6-hydyroxyls of the sugar. This requires that 1- and 6-phosphosugars bind in distinct orientations within the active site, as first revealed by crystal structures of PMM/PGM from *Pseudomonas aeruginosa.*^4^ Another challenge is the required 180° reorientation of the reaction intermediate that occurs in between the two chemical steps, which is known to occur “on enzyme” in several members of the family.^5,6^ Adding to the complexity of recognition, certain subgroups of the superfamily have dual substrate specificity, such as the PMM/PGMs that can effectively utilize both glucose and mannose-based substrates.^7^ Moreover, detailed biophysical studies of several enzymes in the superfamily have established that loss of covalent modification of the catalytic serine by phosphorylation increases the flexibility of the polypeptide backbone,^8–11^ a phenomenon that may facilitate the release and rebinding of the bisphosphorylated sugar intermediate during the course of the reaction.^9^ Overall, the catalytic mechanism of these enzymes demands a robustly designed active site that can accommodate different ligand binding orientations, recognize varying types of sugars and number of phosphorylation sites, and enable the reorientation of the intermediate in the midst of the catalytic cycle.

To further characterize the various enzyme states involved in this unique catalytic mechanism, we obtained multiple high resolution crystal structures of PGM from the plant pathogen *Xanthomonas citri* (XcPGM).^3^ Favorable experimental characteristics of XcPGM crystals enable systematic, detailed structural comparisons of specific enzyme states, including the apo-enzyme, complexes with 1- and 6-phosphosugars, the bisphospho-intermediate, and glucose- and mannose-based sugars. Each ligand complex was characterized with both the phosphorylated and unphosphorylated states of the enzyme, for a total of twelve structures at cryogenic temperatures [Fig. 1(b)]. In addition, we present three high resolution structures of XcPGM from X-ray data collected at room temperature (RT). This study was carefully planned to eliminate potential structural impacts that might arise from differences in crystallization conditions, crystal packing, diffraction source, or data collection/refinement protocols. As a result, we are able to assess subtle structural features in our comparisons and reveal structural snap shots in unprecedented detail along the catalytic cycle of the enzyme.

## RESULTS

### Preparation of different enzyme states

XcPGM was selected for the analyses herein for several reasons. First, the high resolution diffraction of its crystals makes it ideal for detailed structural analyses: previously determined structures of XcPGM were reported at 1.27 Å (apo-enzyme, PDB ID 5BMN) and at 1.85 Å in complex with glucose 1-phosphate (G1P) and G16P (PDB ID 5KLO and 5BMP, respectively).^3^ The high-resolution diffraction and notable mechanical stability of these crystals are likely associated with the observed tight packing arrangement of molecules in the unit cell and their relatively low solvent content (43%) (Fig. S1). The robustness of the crystals also enabled collection of X-ray diffraction data at RT to resolutions near 2.0 Å, which has not been possible for other enzymes in the superfamily. In addition, unlike many related enzymes, XcPGM does not crystallize in high salt (Materials and Methods section).^3^ This greatly facilitates the formation of XcPGM-ligand complexes, as binding of phosphosugars is impeded by high ionic strength. Finally, protocols for preparing phosphorylated and unphosphorylated states of XcPGM were developed based on our previous experience with related enzymes.^11^ Together, these factors enabled an exploration of multiple variables in this system.

The multiple enzyme states characterized (Table I and Fig. S2) include two major comparisons: (1) apo-enzyme vs ligand complexes and (2) phospho- vs dephospho-enzyme (P and deP) representing the active and inactive forms of the enzyme. Within the ligand complexes, we further explored three other variables: 1- vs 6-phospho sugars (substrate/product), glucose vs mannose recognition, and binding of the intermediate G16P. (For this study, we define 1-phosphosugars as substrates and 6-phosphosugars as products, although due to the reversibility of the reaction, either designation is technically correct). While only the phosphoglucomutase activity of XcPGM has been experimentally verified,^3^ it is likely that the enzyme also has phosphomannnomutase activity, based on its sequence homology with the PMM/PGM subgroup of the superfamily.^1^ We therefore included studies with mannose 1-phosphate (M1P) and mannose 6-phosphate (M6P). As a final category, we obtained and analyzed three RT X-ray data sets of XcPGM, as apo-enzyme (both P and deP) and in a complex with glucopyranosyl-1-methyl phosphonic acid (G1CP), a substrate analog. To minimize any structural impacts resulting from differences in crystallization conditions, crystal packing, or data collection/refinement protocols, all structures herein were determined from crystals grown for this study, as described in Materials and Methods (previously deposited structures of XcPGM were not used). High resolution limits of the datasets ranged from 1.35 to 2.05 Å resolution (Table S1).

**TABLE I.** Overview of X-ray data sets collected. For ligand abbreviations, see text. Cryogenic data sets were collected at −170 °C and RT data sets at 25 °C. Coordinate error calculated by Phenix.^60^ ADP = atomic displacement parameters.

	State no.	Ligand	*d_min_* (Å)	Mean ADP (Å^2^)	*R*	*R_free_*	Coord. error (Å)	PDB ID
CRYO								
Apo-P	1	…	1.44	20.5	0.1708	0.2076	0.17	6NN2
Apo-deP	2	…	1.50	27.0	0.1722	0.2054	0.21	6NN1
Phospho-complexes	3	G1P	1.57	22.9	0.1699	0.2062	0.19	6NNO
	4	M1P	1.61	19.1	0.2070	0.2707	0.29	6NOQ
	5	G6P	1.45	19.7	0.1782	0.2104	0.21	6NNS
	6	M6P	1.41	20.8	0.1795	0.2131	0.23	6NP8
	12	G16P	1.46	20.0	0.1691	0.2071	0.16	6NNU
Dephospho-complexes	8	G1P	1.35	20.9	0.1743	0.2028	0.19	6NNN
	9	M1P	1.73	27.3	0.1905	0.2488	0.35	6NOL
	10	G6P	1.50	24.0	0.1792	0.2112	0.19	6NNS
	11	M6P	1.38	20.0	0.1913	0.2221	0.21	6NPX
	7	G16P	1.45	21.7	0.1689	0.2078	0.17	6NNT
		X1P	1.45	16.6	0.1773	0.2147	0.18	6NQH
RT								
Apo-P	13	…	1.90	39.9	0.1695	0.2053	0.22	6NQF
Apo-deP	14	…	1.85	35.5	0.1607	0.1873	0.19	6NQE
Complex	15	G1CP	2.05	37.9	0.1823	0.2529	0.39	6NQG

Careful handling was needed to obtain crystals of certain enzyme states (Materials and Methods). XcPGM purifies as a mixture of P and deP enzyme,^11^ but initial data sets collected from crystals grown at 18 °C showed that the catalytic serine, Ser97, was unphosphorylated. To obtain phospho-enzyme crystals, the purified protein was phosphorylated with G16P prior to crystallization and crystals grown at 4 °C to limit spontaneous hydrolysis of the phosphoserine.^11^ With regard to ligand complexes, formation was routine for crystals stored in liquid nitrogen and subsequently used for cryogenic data collection. Clear electron density was observable for all ligands in Polder omit maps^12^ calculated from the cryogenic data sets (Fig. S3). However, initial RT data sets had no density for ligands, despite using the same soaking conditions successful for the cryo-crystallography experiments. After multiple failures, we considered the possibility that catalysis was occurring in the crystals at room-temperature. To test this, the nonhydrolysable substrate analog G1CP was utilized in soaks, resulting in clear density for the ligand in electron density maps (Fig. S3).

### Apo-enzyme in its active and inactive state

**FIG. 2.**
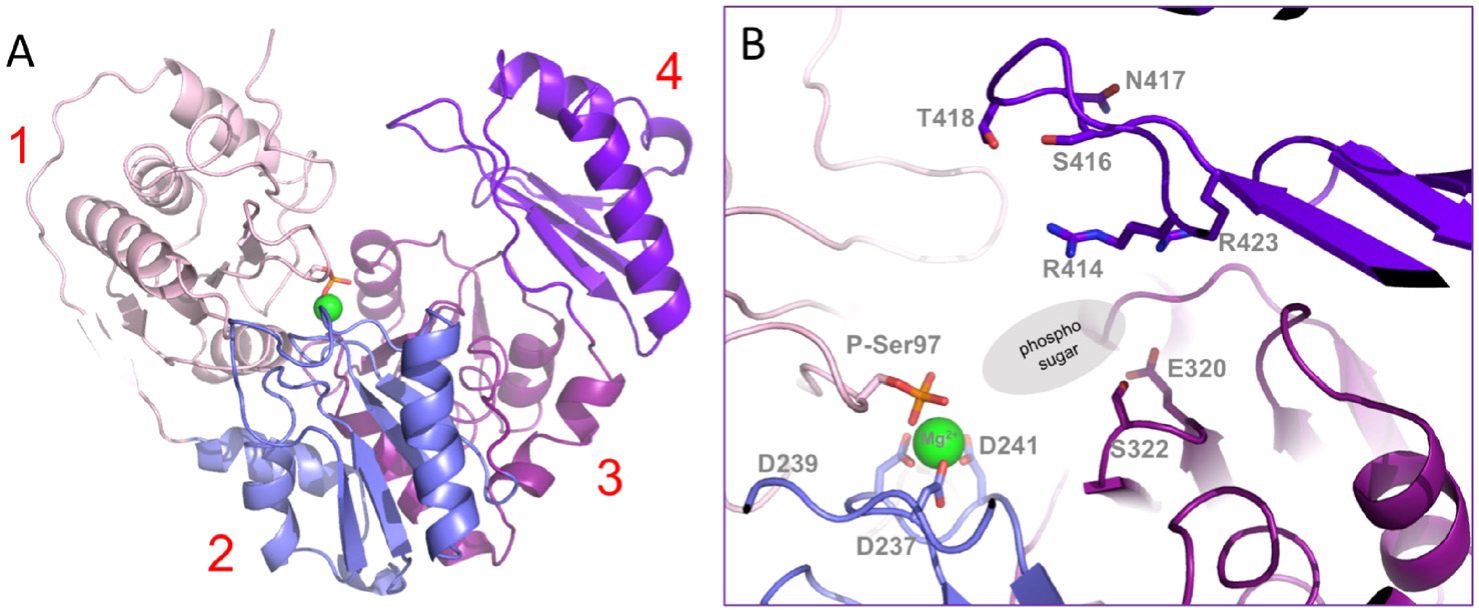
(a) The crystal structure of XcPGM (apo-P) colored by domain [(1)–(4); see labels)]. Phosphoserine 97 is highlighted as sticks and the bound Mg^2+^ ion is a green sphere. (b) A close-up view of the active site, highlighting key functional loops. Residues with roles in catalysis and ligand binding are highlighted in sticks. Colors as in (a). The general vicinity of the phosphosugar binding site is indicated by gray oval.

The structure of apo-XcPGM was determined with both the phosphorylated (E_P_; active) and unphosphorylated (E_deP_; inactive) states of the catalytic serine. Diffraction limits of the two data sets were similar at 1.44 and 1.50 Å for the E_P_ and E_deP_, respectively (states^1,2^ in Table I). Structures were solved by molecular replacement using the coordinates of the previously published apo-enzyme (PDB ID 5BMN).^3^ Like other enzymes in the superfamily, XcPGM has four domains of approximately equal size, arranged in an overall heart-shape [Fig. 2(a)]. The active site is located in a large central cleft at the confluence of its four structural domains, and involving >60 residues. Within this cleft, four loops (one from each domain) have conserved functional roles across the enzyme superfamily (for the detailed review see Ref. 1) In domains 1–4, these loops are: (i) the phosphoryl transfer loop (residues 95–99) including phosphoserine 97; (ii) the metal-binding loop with its three coordinating aspartates (residues 237–241); (iii) a sugar-binding loop that includes Glu320 and Ser322; and (iv) the phosphate-binding loop (residues 414–423) that interacts with the phosphate group of the ligands. Figure 2(b) shows a close-up view of these regions in the active site of XcPGM.

**FIG. 3.**
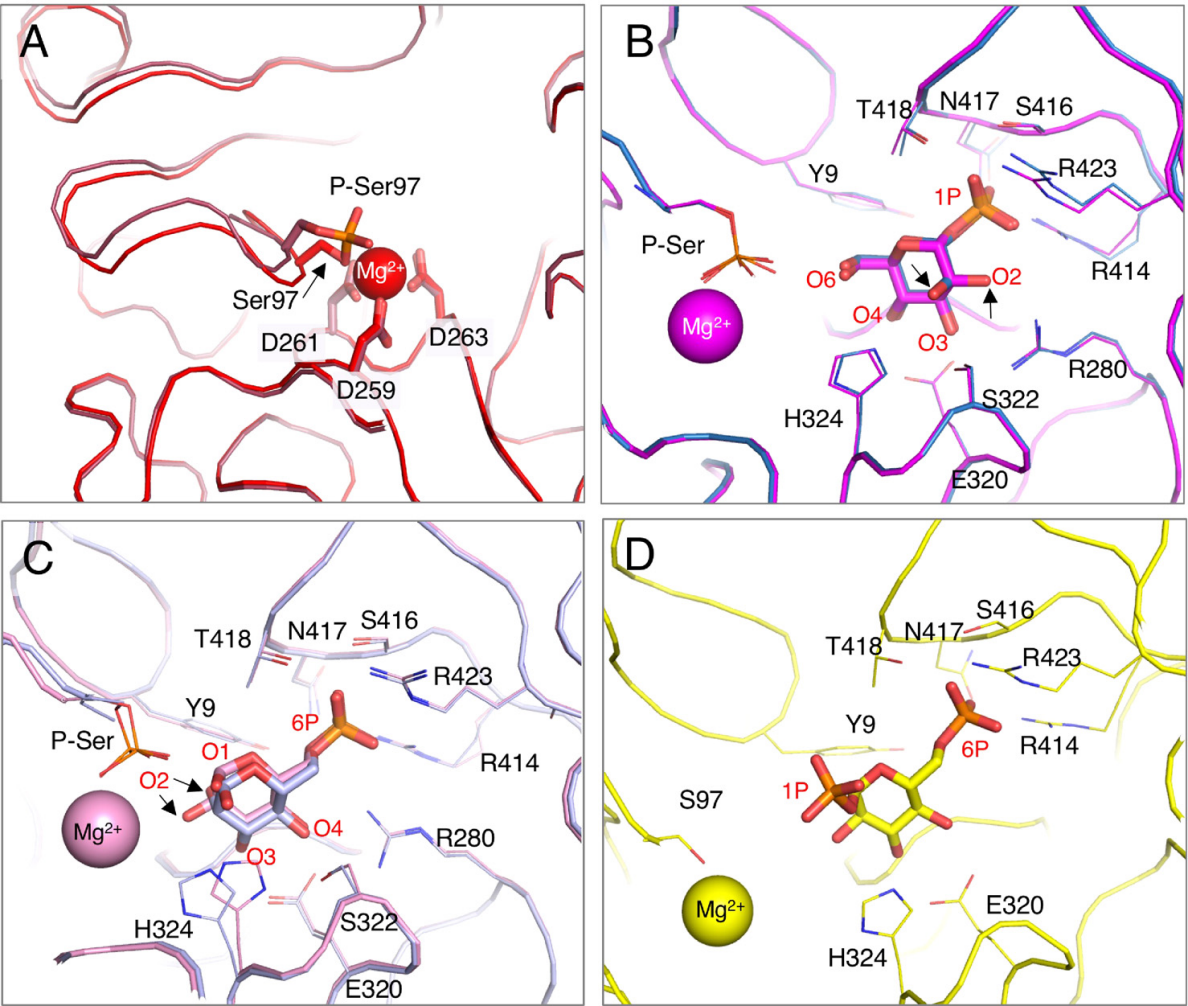
Close-up views of the XcPGM active site in various enzyme states. (a) Loop (i) in E_P_ (dark red) and Ed_eP_ (red), showing the impact of phosphorylation in this region. The bound metal (sphere) and its three coordinating aspartates are also shown. (b) A superposition of the two E_P_:S complexes with G1P (magenta) and M1P (blue). Residues involved in ligand contacts or with roles in catalysis are shown as sticks and labeled; arrows highlight the two different positions of O2 in glucose and mannose. (c) A superposition of the two E_P_:S complexes with G6P (pink) and M6P (light blue). (d) A view of G16P in the E_deP_:intermediate complex (yellow). In certain structures, side chains with multiple conformers have been omitted, for clarity.

Overall, the structures of E_P_ and E_deP_ are very similar, with a C_α_ root-mean-square deviation (RMSD) between polypeptide backbones of 0.26 Å (Table S2). A small shift in the backbone between these two structures is evident in loop (i) and several other regions near the site of phosphorylation [Fig. 3(a)]. In E_deP_, the sidechain hydroxyl of the serine acts as a ligand for the Mg^2+^ ion, along with three sidechain oxygens from the aspartates in loop (ii). In E_P,_ one of the phosphate oxygens takes the place of the serine hydroxyl and coordinates the metal along with the aspartates in loop (ii). A structural shift of loop (i) between E_P_ and E_deP_ has not been observed in other enzymes in the superfamily, although only a few have been crystallized in both states.^9,10,13^ It remains to be seen whether changes in loop (i) related to phosphorylation are a common feature in the superfamily.

### Phospho-enzyme complexes with substrates

Structures of phospho-XcPGM in complex with substrates (S) G1P and M1P were obtained at 1.57 and 1.61 Å resolution (states^3,4^ in Table I). Relative to the apo-enzyme, the E_P_:S complexes show a conformeric change in loop (iv) of domain 4. This results in an overall RMSD with apo-enzyme of ∼0.6 Å (Table S2), while the two E_P_:S complexes are quite similar to each other (RMSD 0.29 Å). Loop (iv) rotates inward, toward the active site, positioning residues in this region to interact with the bound substrate. As expected (and seen in the previous G1P complex of XcPGM^3^), both G1P and M1P bind such that their phosphate group interacts with loop (iv) [Fig. 3(b)]. Interactions to the 1-phospho group of both G1P and M1P are made by residues Arg414, Ser416, Asn417, Thr418, and Arg423 (Table S3) with at least seven direct hydrogen bonds/salt bridges per complex, and an additional water-mediated interaction between the phosphate group and Tyr9/Asn417. These extensive interactions are consistent with their proposed role as an “anchor” for phosphosugar recognition in the enzyme superfamily.^4^ Anchoring of the phosphate group of the substrate by loop (iv) serves to position O6 in the vicinity of phosphoserine 97, as needed for phosphoryl transfer [Fig. 3(b)]. (Ser97 is ∼5 Å from O6 in the enzyme-ligand complexes suggesting that a small conformational adjustment of the protein would be needed for catalysis to proceed).

In addition to the phosphate contacts, other interactions are made between the protein and the hydroxyl groups of the substrates [Fig. 3(b)]. These include contacts to O3 and O4 from the side chains of Glu320 and Ser322, and the backbone amide of His303. These interactions, particularly the bidentate interaction by a glutamate, are conserved in the enzyme superfamily.^4,14^ Contacts made with O2 of the hexose reflect its differing stereochemistry in glucose and mannose (equatorial vs axial). In the G1P complex, Arg280 makes a bidentate interaction with O2 and O3. In the M1P complex, the contact between Arg280 and O3 is maintained, but Ser322 now contacts both O2 and O3 of the mannose. Thus, differential side chain contacts accommodate the differing stereochemistry of O2 in the substrates of XcPGM. Another nearby residue in the active side is His324 (<4 Å from O6), which is a candidate for the general base in the reaction, based on studies in related enzymes.^15^ Enzyme contacts with the substrate analog G1CP in a 2.05 Å resolution RT data set, state,^15^ are very similar to those with G1P (Table S3).

### Phospho-enzyme complexes with products

Structures of phospho-XcPGM in complex with the products (P) glucose 6-phosphate (G6P) and M6P were obtained at 1.45 and 1.41 Å resolution (states^5,6^ on Table I). Similar to the E_P_:S complexes, the E_P_:P complexes also show a closure of loop (iv) relative to apo-enzyme (RMSD ∼ 0.6 Å with apo-enzyme and 0.22 Å between the G6P and M6P complexes; Table S2). Contacts to the 6-phosphate group are made by the same residues in loop (iv) as in the E_P_:S complexes: Arg414, Ser416, Asn417, Thr418, and Arg423 [Fig. 3(c)]. These invariant contacts to the phosphate group, seen in complexes with both 1- and 6-phosphosugars, are consistent the enzyme mechanism, whereby a ∼180° reorientation of the bisphosphorylated intermediate occurs in the midst of the catalytic cycle (Fig. 1). The rotation axis is approximately defined by O5 and the midpoint between O3 and O4 [compare Figs. 3(b) and 3(c)]. In the E_P_:P complexes, anchoring of the 6-phosphate group of the product by loop (iv) positions O1 (rather than O6 as in the E_P_:S complexes) near phosphoserine 97, as would be found after the second phosphoryl transfer in the catalytic cycle [Fig. 1(a)].

Due to the alternate binding orientation of the sugar ring in the E_P_:P complexes, O3 and O4 exchange places compared to their positions in the E_P_:S structures. This exchange allows the same enzyme residues to contact these two hydroxyls: the side chains of Glu320 and Ser322, and the backbone amide of His303 [Fig. 3(c)]. This is possible because both O3 and O4 have equatorial stereochemistry in glucose and mannose,^4^ allowing them to essentially switch places when the sugar ring is flipped by 180°. In contrast, the 180° reorientation places O2 in in a very different position in the active site of the E_P_:P complexes compared to the E_P_:S complexes. In neither case, with G6P or M6P, direct enzyme contacts to O2 are observed. Thus the active site of XcPGM is permissive for multiple positions of O2 in the E_P_:P complexes, but lacks specific contacts. It is interesting to note that despite the different binding orientations of the ligands, the protein structures in the E_P_:P complexes are quite similar to the E_P_:S complexes (RMSD of 0.16–0.39 Å depending on structures compared; Table S2).

### The dephospho-enzyme complex with the intermediate

A structure of XcPGM in its dephosphorylated state bound to the G16P intermediate (I) was determined at 1.45 Å resolution. The E_deP_:I complex (state^7^ on Table I) represents one of two possible orientations for binding of G16P necessary to complete the catalytic cycle (Fig. 1). These are: (1) with its 6-phospho group near loop (iv), as seen here [Fig. 3(d)]; and (2) with its 1-phospho group near loop (iv), which is not observed. Both binding orientations must occur during the catalytic cycle depending on whether the first or second phosphoryl transfer needs to or has already taken place, although only the first has been observed in crystal structures in the superfamily.^16^ Other catalytically relevant states involving E_deP_:I, which are not characterizable by crystallography, include the two phosphoryl transfer steps, shown for a related enzyme to proceed through a concerted S_N_–2-like mechanism, with a loose, metaphosphate-like transition state.^17^ Another is the dynamically reorienting G16P present in between phosphoryl transfer steps [Fig. 1(a)], which has been detected by single turnover kinetics in the case of *P. aeruginosa* PMM/PGM.^6^

The E_deP_:I complex reflects a productive step in the catalytic cycle, where the enzyme has donated its phosphoryl group to substrate, creating intermediate, but has not yet transferred a phosphoryl group back to the enzyme to create product. The enzyme adopts a closed conformer of loop (iv) similar to the E_P_:S and E_P_:P complexes. Multiple interactions [Fig. 3(d)] are found between the protein and G16P, including contacts between loop (iv) and the 6-phosphate group, as observed in the E_P_:P complexes. Also conserved are contacts with the O3 and O4 hydroxyls by Glu320 and His303 (see Table S3 for a complete list). No contacts are made between the enzyme and the 1-phospho group of G16P, consistent with its participation in the phosphoryl transfer reaction. Overall, the E_deP_:I complex is most similar to the E_P_:S and E_P_:P complexes (RMSD 0.27–0.35 Å; Table S2).

The enzyme-ligand contacts in the E_deP_:I complex seen here, and previously with *P. aeruginosa* PMM/PGM,^16^ appear to reflect a high-affinity binding interaction with G16P, somewhat in contradiction to the enzyme mechanism that requires a reorientation of the intermediate to complete the catalytic cycle [Fig. 1(a)]. As noted above, hydrogen-deuterium exchange by mass spectrometry, NMR, and various biochemical studies of other enzymes in the superfamily has suggested that E_deP_ has increased flexibility of its polypeptide backbone relative to E_P._^8,9,11,18^ These flexibility changes, which have not been apparent in crystal structures, have been proposed to facilitate the release and reorientation of G16P.^9,11^

### Nonproductive enzyme complexes

Also as part of this study, we characterized a series of “off pathway” complexes of XcPGM. These include E_deP_ with a bound substrate or product (states^8–11^ on Table I) as well as an E_P_:I complex (state^14^) also previously determined.^3^ None of these are part of the productive catalytic cycle, but may occur, for example, if the enzyme binds the ligand after losing its phosphoryl group from hydrolysis. Similar to the E_P_:S and E_P_:P complexes, the E_deP_ complexes also show a closure of loop (iv) relative to apo-enzyme. Also, some small structural shifts are present in/near loop (i) that appear to be related to the lack of phosphorylation of Ser97, as noted in the apo E_deP_ structure [Fig. 3(a)]. Overall, however, the E_deP_ complexes are highly similar to their counterparts with phospho-enzyme (pairwise RMSDs of 0.16–0.25 Å; Table S2), so we omit a detailed discussion of their enzyme-ligand contacts (see Table S3 for summary). The nonproductive complexes are included as part of the ensemble analysis in a following section (Functional categorization of structures by PCA).

The E_P_:I structure is a somewhat distinct unproductive complex, as it has an extra phosphate group in the active site, due to the phosphorylated state of Ser97 and the two phosphate groups of the intermediate. This complex could occur if free (unbound) G16P happened to encounter and bind to phospho-enzyme in solution, but this would likely be a rare event since the intermediate typically remains associated with enzyme during the catalytic cycle.^5,6^ Despite the additional phosphate, G16P in the E_P_:I complex binds in a generally similar orientation to that observed in the E_deP_:I complex, with small adjustments in the enzyme-ligand contacts (Table S3). A similar complex of E_P_ with G16P was observed with *P. aeruginosa* PMM/PGM.^16^

### Insights into the conformational ensemble of XcPGM from RT crystallography

In addition to the structures described above, all from diffraction data collected at cryogenic temperatures, we obtained three RT data sets on XcPGM (states^13–15^ on Table I). These structures were initially sought to provide information on the conformational ensemble of the protein through polysterism analysis, as done in other systems.^19–21^ However, use of the software qFit 2.0 (Ref. 20) with our data yielded multiple structures with distinct sets of side chain conformers but nearly indistinguishable R_free_ values (data not shown). This precludes identification of a single pathway of coupled side chain conformers (via the program CONTACT^19^). Hence this approach was not useful for our system, as also reported for a different enzyme.^22^

**FIG. 4.**
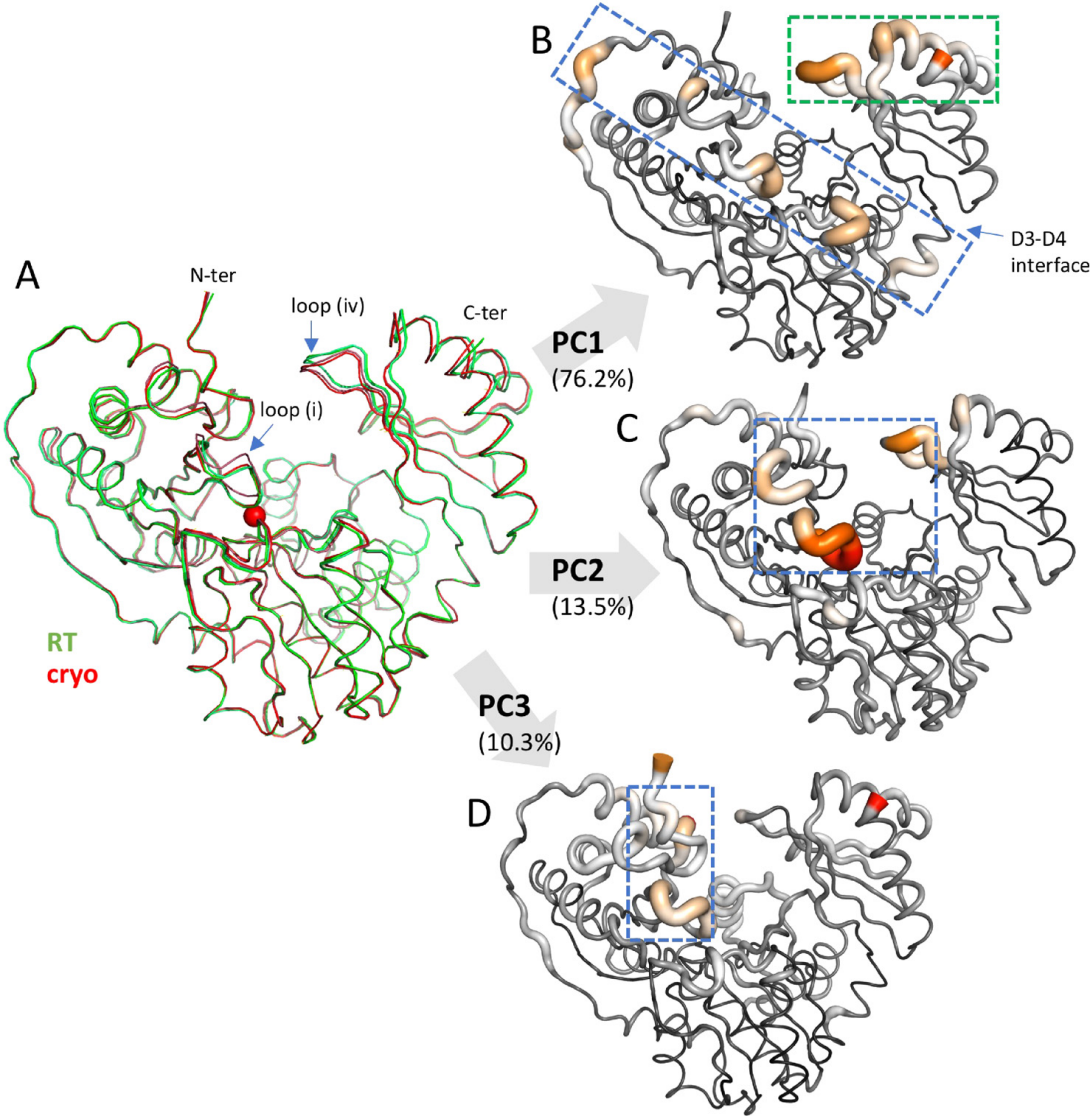
PCA of an apo-enzyme ensemble of XcPGM comprised of two cryo and two RT structures. (a) A superposition of the four apo-enzyme structures, states.^1,2,13,14^ (b)–(d) Individual residue contributions for the first three principal components plotted on the structure. Magnitude of contribution is indicated by color (low, black; high, red) and tube width. The contribution of each component to the overall variance is indicated in parentheses. Dashed boxes highlight groups of covarying residues (see the text).

As an alternative for gaining structural insights from the RT data sets, we used principal component analysis (PCA) to analyze the atomic coordinates of E_P_ and E_deP_ XcPGM determined at both cryogenic and room temperatures [for superposition see Fig. 4(a)]. PCA is a widely used statistical method useful for emphasizing variation and identifying strong trends within a dataset. It has found utility in the characterization of protein ensembles from NMR^23,24^ and molecular dynamics studies.^25–27^ However, PCA is not commonly used in crystallographic comparisons, which typically involve fewer structures. The use of PCA in coordinate comparisons may have also been limited by the complexity of early implementations,^28^ but the availability of the R^29^ software package Bio3D^30^ makes PCA routine for biomolecules (see supplementary material File S1). The four apo-enzyme structures (states^1,2,13,14^) were used to create an ensemble; pairwise RMSDs for these structures range from 0.23 to 0.50 Å (Table S2). As implemented in Bio3D, structures were automatically aligned and PCA was performed on the covariance matrix of C_α_ coordinates (Materials and Methods). The first three components (PC1–PC3) account for 100% of the variance in the ensemble. Individual contributions (i.e., loadings) of each residue to PC1–PC3 were mapped onto the structure [Figs. 4(b)–4(d)]. Each principal component comprises information on atomic variance across the entire structure, such that the highlighted residues exhibit co-varying structural changes regardless of their proximity in space. Moreover, the variations in each principal component are independent from the others, even though sometimes the same residues are involved in more than one component.

PCA of the cryo-RT ensemble reveals regions of XcPGM with correlated structural changes (Fig. 4). For instance, in PC1 [Fig. 4(b)], it can be seen that variations in domain 4 (green box) are coupled to a swath of structural changes across the protein, including loop (i) and other residues in domains 1 and 3 (blue box). In PC2 [Fig. 4(c)], the co-varying regions are more localized (blue box), with the greatest variations in loop (i), another nearby loop in domain 1, as well as loop (iv). Finally, in PC3 [Fig. 4(d)], covariation is seen between loop (i) and a different region of domain 1 (blue box).

Several advantages of PCA are apparent from this inquiry. While some regions of XcPGM highlighted by PCA have noticeable variation in the structural superposition [e.g., loops (i) and (iv) in Fig. 4(a)], other areas evident from PCA are more difficult to discern in the superposition due to high similarity of the structures. More importantly, PCA provides information on correlated structural variations, which cannot be gleaned from the superposition. Some of these, such as PC1, involve a large number of residues, spanning multiple domains of the enzyme and connecting key active site loops with residues on the periphery of the structure and in domain interfaces. Such groupings could potentially indicate residue networks with catalytic relevance. Other components, like PC2, show a different type of co-variation between key active site loops (independent of those seen in PC1), suggesting more than one type of coupled motion in these loops. Finally, we find that the structural variations highlighted in the cryo-RT ensemble are reflected in the variations observed between the different enzyme states at cryogenic temperatures. For instance, the conformational variability of loop (iv) is clear (and highlighted by PCA), even though the cryo-RT ensemble includes only apo-structures. This suggests that PCA of structures determined at cryogenic and room temperatures can provide insight into biologically relevant protein conformers, without the need for more complicated computational analyses.

### Functional categorization of structures by PCA

**FIG. 5.**
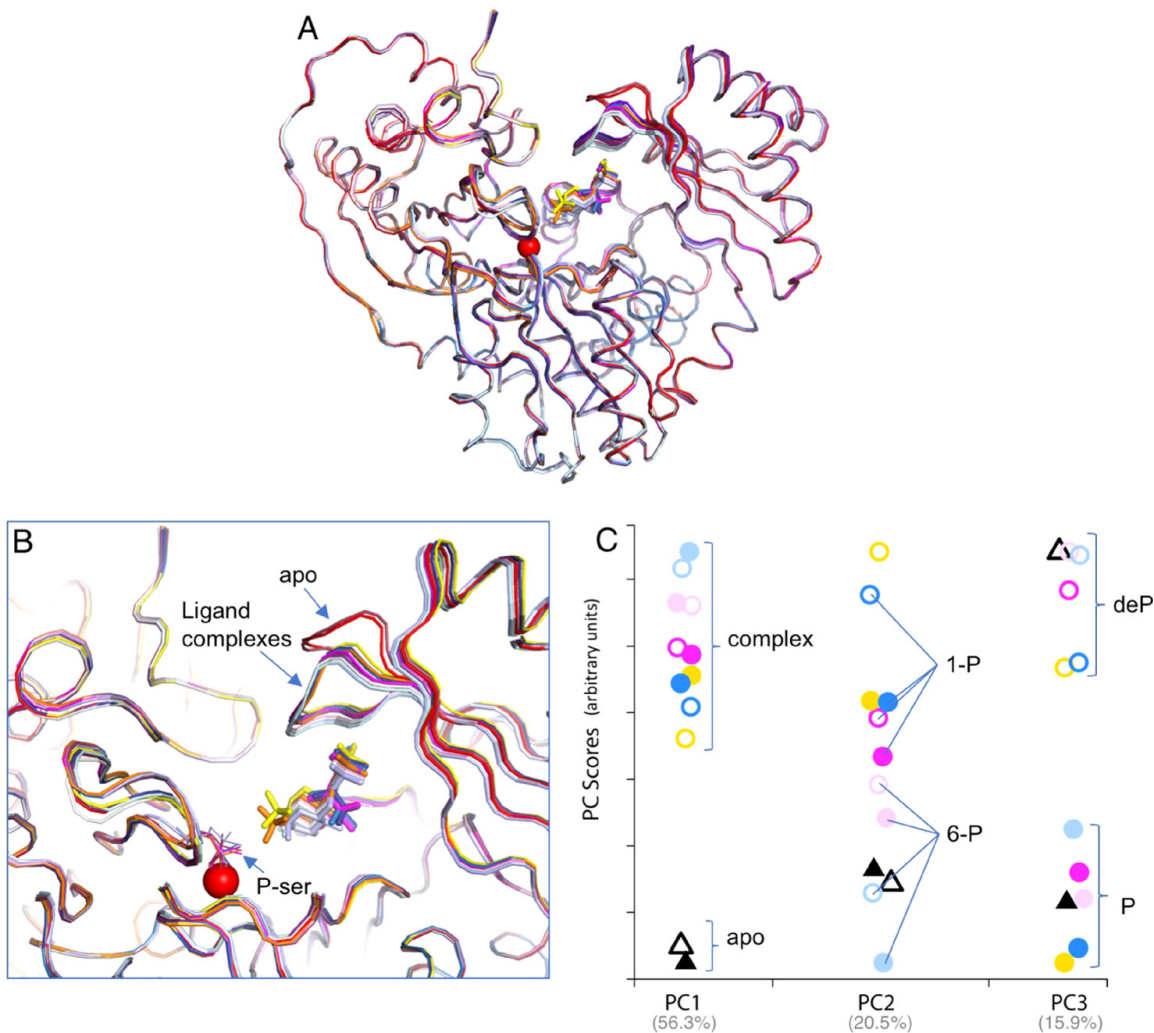
Functional groupings of XcPGM enzyme states determined *a priori* from PCA. (a) A superposition of the 12 cryogenic XcPGM structures and their bound ligands. The metal ion is shown as a red sphere. (b) A close-up view of the active site from the superposition in (a). Colors of structures are as in Fig. 3, with addition of the E_P_: intermediate complex with G16P (orange), and the dephospho-enzyme ligand complexes with G1P, G6P, M1P, and M6P in purple, white, dark blue, and cyan, respectively. (c) A scatter plot displaying scores of the various XcPGM structures for the first three principal components of the data set. Apo structures are shown as black triangles; ligand complexes as circles. Phospho- and dephospho-enzyme structures are solid and open symbols, respectively. Complexes with glucose-based sugars are in shades of pink, mannose-based sugars in blue, and G16P complexes in yellow. Bright colors are E_P_:S complexes (1-phosphosugars) and pastels are E_P_:P complexes (6-phosphosugars). Random noise (jitter) was added to the x-dimension to separate points for ease of visualization; different components have been normalized to the same scale on the vertical axis.

We also investigated the utility of PCA to probe relationships among the various enzyme-ligand complexes. As noted in previous sections, the various XcPGM structures are highly similar based on traditional measures such RMSD (Table S2) or as seen in structural superpositions [Figs. 5(a) and 5(b)]. While variability is evident in loop (iv), for instance, when comparing the apo-enzyme to ligand complexes, structural differences between the ligand complexes (e.g., E_P_:S vs E_P_:P) are not obvious. In general, assessment of subtle differences between crystal structures is complicated by the coordinate uncertainty inherent to crystallographic models,^31,32^ and may also be affected by other errors/biases in the structural models or related to the model building process.^33,34^ PCA helps overcome these potential complications, since it highlights the large trends or patterns in the data. It is also simple to determine the significance of the individual components through their percent contribution to the total variance of the data set.

To assess the possibility of subtle structural changes relevant to the different steps of the catalytic cycle, we employed PCA reduction using the C_α_ coordinates of the 12 cryogenic XcPGM structures. All structures were included in order to probe the different variables represented within the ensemble (e.g., apo-enzyme vs ligand complex, substrate vs product, glucose vs mannose, and P vs deP structures). A scatter plot illustrates separation of the structures in the first three components (PC1, PC2, PC3) of the data set [Fig. 5(c)]. Together, these comprise 92.7% of the variance in the ensemble, with contributions of 56.3%, 20.5%, and 15.9%, respectively. The scores (y-axis) indicate separation of the structures along these components. In PC1, a distinct separation is seen between the two apo structures (triangles) and the ligand complexes (circles). This variation is the most significant in the data set, consistent with the obvious conformational change of loop (iv) upon ligand binding. In PC2, the scoring of the structures is dramatically different (compare positions of analogous symbols), showing the independence of this component from PC1. In PC2, the structures are distributed more evenly by score, with the 1-phospho vs 6-phospho structures, and especially the mannose-based sugars (blue), tending to fall at different ends of the distribution (bold vs pastel colors). In PC3, two clusters are again seen, which separate clearly according to the phospho- and dephospho-enzyme states (open vs solid symbols). For an animated view of the PCA plots in 3D, see Fig. S4.

Here we find that despite the subtle structural differences within this ensemble, and in the absence of any functional input, PCA successfully clusters the XcPGM ensemble into meaningful groups. In this case, as the roles of the structures along the catalytic cycle of XcPGM are known, we can confirm that the separation by PCA is correlated with the enzyme state. However, PCA is equally applicable to systems where functional information is not available or pertinent, such as protein complexes with inhibitors or other artificial ligands. We also show that PCA is useful despite the overall structural similarity of the ensemble. Even at the relatively high resolution of diffraction in the data sets in this study, the coordinate errors of the structures range from ∼0.2 to 0.4 (Table I), and are often similar to the pairwise RMSDs of the structures (Table S2). PCA thus helps overcome a traditional problem in crystallography of assigning significance to structural differences by identifying them as part of a co-varying group. Finally, even though only the C_α_ coordinates of the proteins were used in the alignment, PCA was still able to cluster the structures according to enzyme state, showing that this information is encoded in the structures without considering side chain positions. However, similar analyses could be done on a per atom basis (assuming matching sets of atoms), if desired, and would likely reveal additional information.

## DISCUSSION

The high resolution structures of XcPGM determined in this study populate most of the observable structural states along the catalytic cycle of the enzyme, and illustrate key themes in enzyme mechanism and substrate recognition in the α-D-phosphohexomutase superfamily. In the E_P_:S and E_P_:P complexes, the substrates and products are found to occupy the same ligand binding site and utilize the same residue interactions with protein, despite the differing orientations of their sugar ring. These structures are consistent with the proposed enzyme mechanism,^1^ whereby a 180° flip of the intermediate occurs, following the initial phosphoryl transfer to substrate and prior to the subsequent phosphoryl transfer from the intermediate to the enzyme. Including the structures herein, crystallographic studies have helped confirm this mechanism in three members of the superfamily,^4,14^ further suggesting it as a common feature of these ubiquitous enzymes.

Enzyme-ligand interactions of the various XcPGM enzyme states are also revealed in great detail, including the conserved residues in the phosphate-binding site, loop (iv), as well as the residues responsible for contacts with the O3/O4 hydroxyls. Both of these regions are highly conserved in sequences of the α-D-phosphohexomutases, although some variations within enzyme subgroups have been noted.^35,36^ The exchangeability of the O3/O4 interactions is dependent on the equatorial stereochemistry of these hydroxyl groups, as found in glucose or mannose, but not in related sugars such as galactose. The conserved O3/O4 contacts are consistent with the substrate preferences known for entire superfamily, which also uses glucose-derived substrates such as glucosamine and N-acetylglucosamine, where the varying substituents are confined to the 2-position of the sugar.^1^

Comparisons of the polypeptide backbone in the various XcPGM structures reveal conformational changes in two active site loops, related in one case to ligand-binding, loop (iv), and, in the other, to a change in the phosphorylation state of the catalytic serine in loop (i). While the former has been seen in other enzymes in the superfamily,^14,37^ direct structural changes related to phosphorylation have not been characterized previously, although other effects of this covalent modification have been noted.^8,10,11,18^ Thus the XcPGM structures add to the types of conformational variations associated with the catalytic cycle of the α-D-phosphohexomutases. Unlike some other enzymes in the superfamily,^4,38,39^ conformational variability of domain 4 is not notable in the XcPGM structures described here, perhaps due to the tight packing in the crystal lattice.

Also as part of this study, we utilized the statistical method of PCA to analyze two different ensembles of XcPGM structures. PCA is a quick and convenient way to highlight structural differences, even when these may difficult to assess by traditional measures like RMSD. Although trivial in some cases, manual inspection of structural superpositions can quickly become overwhelming when large proteins or many subunits are involved. Here, we illustrate two uses of PCA reduction in coordinate analysis: to highlight regions of co-varying structural changes in XcPGM (using the cryo-RT ensemble) and to cluster the structures into related groups (within the enzyme state ensemble). It is easy to think of other types of protein ensembles where different types of relationships could be explored, such as domain rotations,^40,41^ packing of oligomers,^42^ impacts of mutations,^43^ or binding of fragments for drug discovery.^44^

As noted above, PCA is commonly used to analyze structural ensembles resulting from NMR studies^23,24^ and molecular dynamics simulations.^25–27^ PCA and similar statistical methods have found additional uses in crystallography, most frequently in the analysis of time-resolved X-ray data.^45–48^ Other recent applications include the examination of electron density maps for radiation-induced damage^49^ and to compare microfocus diffraction from different regions of a crystal.^50^ In contrast to these more specialized applications, we emphasize here the straightforward use of PCA for comparing related protein structures, a growing need in the field of structural biology.

PCA of crystallographic coordinate sets has particular utility for the analysis of large structural data sets. For example, X-ray crystallography is increasingly being used to characterize protein-ligand complexes, the numbers of which exceed capacity for detailed study (currently >50 000 protein-ligand complexes in the Protein Data Bank^51^ with nearly 20 000 unique ligands). Because of this, comparative studies tend to focus on obvious structural features (e.g., ligand binding sites) and forego inspection of other regions/areas of the protein, potentially discarding information from uncharacterized functional sites. Finally, even in cases where comprehensive analyses have been conducted, apparent differences between structures may be subtle or exceed the patience of the examiner. Such factors can be an impediment to taking full advantage of available structural data. PCA is well suited to address these challenges, as it provides rapid simplification of coordinate sets into more manageable groupings. Results from PCA can be easily correlated with biochemical properties/phenotypes that allow the functional significance of the results to be further evaluated.

## MATERIALS AND METHODS

### Materials

All chemicals were of reagent grade and purchased from Thermo Fisher Scientific (Waltham, MA) unless otherwise noted. Ligands were purchased from Sigma-Aldrich (St. Louis, MO), with the exception of G1CP, which was synthesized as previously described.^52^

### Protein expression and purification

The gene for XcPGM was commercially synthesized (GenScript) and inserted into the pET-14B vector with N-terminal His6 affinity tag and tobacco etch virus protease cleavage site. The vector was transformed into *Escherichia coli* BL31(DE3) for recombinant expression. *E. coli* cultures were grown at 37 °C in 0.5–1.0 l of LB media, supplemented with 0.1 mg/ml of ampicillin, to an OD600 of 0.8–1.0. Prior to induction with isopropyl 1-thio-β-D-galactopyranoside (final concentration 0.4 mM), cultures were cooled at 4 °C for at least 30 min. Cells were induced for ∼18 h at 18 °C. Cell pellets were collected by centrifugation and flash frozen in liquid N_2_ and stored at −80 °C. Protein was purified to homogeneity via an N-terminal histidine tag as described.^53^ The purified protein was dialyzed into a solution of 12.5 mM Tris-HCl, pH 8.0, with 50 mM NaCl, and concentrated to ∼11 mg/ml. The purified protein was flash-frozen in liquid nitrogen and stored at −80 °C. Approximately 100 mg of purified protein was obtained from 1 l of cultured cells.

### Crystallization and formation of specific enzyme states

Purified XcPGM was initially screened for crystallization via hanging drop vapor diffusion using the previously published conditions,^3^ but did not yield data collection quality crystals. Several commercial screens were then utilized, including Morpheus 1 and Hampton Screen 1. Optimizations were setup around several hits, and a final condition of 22% PEG 8000, 0.2 M MgCl_2_, 0.1 M HEPES, pH 7.5, was identified and used for all crystals described herein. Crystals typically grew overnight at 18 °C in an unusual morphology, as clusters of hollow rods. Despite the different crystallization conditions, the XcPGM crystals reported here were (Table S1) isomorphous with those published previously.^3^

Crystals of XcPGM grown as above were not phosphorylated at Ser97. To obtain structures of the phospho-enzyme, the protein was pretreated with a molar excess of the activator G16P, as previously described.^11^ Excess G16P was subsequently dialyzed away, and the protein crystallized as above except at 4 °C. (Both the phosphorylated protein and crystals were kept at 4 °C at all times to limit hydrolysis of the phosphoryl group, which occurs more rapidly at higher temperatures in related enzymes^8,11^).

Ligand complexes were obtained by soaking crystals with high concentrations of ligands. Ligand solutions at ∼20 mM were prepared in the crystallization buffer supplemented with or without cryoprotectant (see below). Typically, crystals destined for cryo-crystallography were removed from the drop, dipped quickly into the ligand solution, immediately flash-cooled, and stored in liquid nitrogen. Crystals for room-temperature data collection were soaked in a solution of ligand in crystallization buffer and mounted as below.

Crystals for cryogenic data collection were cryoprotected by adding 25%–30% PEG 3350 (either with or without the ligand) to the crystallization buffer, and were then mounted on Hampton loops/pins. Crystals for room-temperature data collection were mounted in glass capillaries with plugs of crystallization buffer on either side and sealed with wax. Crystals for cryogenic data collection were shipped in a cryo dry shipper to the Advanced Light Source for data collection. Capillaries with crystals for room-temperature data collection were cushioned with glass wool inside conical tubes and shipped with gel packs precooled to 4 °C to the beamline.

### X-ray diffraction data collection and refinement

Diffraction data were collected at a wavelength of 1.00003 Å from single crystals on beamline 4.2.2 of the Advanced Light Source using a Taurus-1 CMOS detector in shutterless mode. To obtain the highest possible resolution data, multiple data sets (2 on average for cryogenic, 2–5 for room-temperature collection) were collected from the same crystal after translating in the beam. Data sets were confirmed to have correlation coefficients greater than 0.95 prior to merging with the x scale of the XDS suite. RT crystals were mounted by hand by attaching the capillary tube to a magnetic base with modeling clay and cryogenic samples were mounted with the beamline ACTOR robot. To mitigate radiation damage, RT crystals were collected for 20° before translating to a fresh area of the crystal and continuing. Analysis of radiation damage was monitored by following the Rd statistic^54^ from XDSSTAT. The data were processed using XDS^55^ and AIMLESS^56^ via CCP4i.^57^ Data processing statistics are in Table S1. Values of *CC*_1/2_ > 0.30 (Ref. 58) and *R*_pim_ (Ref. 59) were used to determine the high resolution cutoff due to the large number of images (1800–3600 per data set) and high redundancy obtained with the shutterless data collection.

Crystallographic refinement calculations were initiated using coordinates of apo-XcPGM (PDB code: 5BMN). Refinement was performed with PHENIX;^60^ progress was monitored by following *R*_free_ with 5% of each data set was set aside for cross validation. The B-factor model consisted of an isotropic B-factor for each atom; Translation/Libration/Screw (TLS) refinement was used as automated in PHENIX. COOT^61^ was used for model building. Structures were validated using MolProbity^62^ and refinement statistics are listed in Table S1. Structural figures were prepared with PYMOL.^63^ Coordinates and structure factor amplitudes have been deposited in the Protein Data Bank (PDB) under the accession numbers listed in Tables I and S1.

### Principal component analysis

PCA was conducted using an in-house script, supplementary material File S1, which is referenced by the commented steps. Structures were loaded into R as PDB files (#Step 2), aligned (#Step 2 and 9), and pairwise RMSD values were calculated (#Step 9) using the read.pdb, pdbaln, and rmsd functions of the Bio3D R package, respectively. Principal components were calculated using the pca.xyz (#Step 11) function of the Bio3D R package. The Scree (cumulative variance) plot was inspected to determine how many components to continue with in the analysis. The scores of structures' xyz coordinates in PC space were plotted in PC pairs (i.e., PC1 vs PC3) to demonstrate clustering of similar structures (#Step 12). Due to the nature of PCA on coordinate-space data, the contribution of each C_α_ atom to a specific principal component is calculated automatically by the pca.xyz function. These values were accessed through the atom-wise loadings (#Step 13 and 14, also see pca.xyz function documentation of Bio3D), mapped to the structure (#Step 15), and visualized in PYMOL.^63^

For additional types of analyses, it is useful to know the organization of the dataframe resulting from PCA. Bio3D's PCA function pca.xyz produces a dataframe consisting of six components named: L, U, z, au, sdev, and mean corresponding to the eigenvalues, eigenvectors (x, y, and z variable loadings), scores of the coordinates on the PCs, atom-wise loadings (normalized eigenvectors), the standard deviations of the PCs, and the means that were subtracted. Of these, primarily the scores (z) and the atom-wise loadings (au) are used. Assuming that the dataframe was named pca.xyz these two variables can be manually accessed with pca.xyz$z and pca.xyz$au in R.

## SUPPLEMENTARY MATERIAL

See supplementary material for additional tables, figures (including an animation of the PCA plot), and script for running PCA on multiple coordinate files.
